# Reintubation of patients submitted to cardiac surgery: a retrospective analysis

**DOI:** 10.5935/0103-507X.20170028

**Published:** 2017

**Authors:** Cíntia Yukie Shoji, Luciana Castilho de Figuereido, Eveline Maria Calixtre, Cristiane Delgado Alves Rodrigues, Antonio Luis Eiras Falcão, Pedro Paulo Martins, Ana Paula Ragonete dos Anjos, Desanka Dragosavac

**Affiliations:** 1 Departamento de Cirurgia, Universidade Estadual de Campinas - Campinas (SP), Brasil.

**Keywords:** Noninvasive ventilation, Cardiac surgery, Intubation, Mortality, Respiratory insufficiency

## Abstract

**Objectives:**

To analyze patients after cardiac surgery that needed endotracheal reintubation and identify factors associated with death and its relation with the severity scores.

**Methods:**

Retrospective analysis of information of 1,640 patients in the postoperative period of cardiac surgery between 2007 and 2015.

**Results:**

The reintubation rate was 7.26%. Of those who were reintubated, 36 (30.3%) underwent coronary artery bypass surgery, 27 (22.7%) underwent valve replacement, 25 (21.0%) underwent correction of an aneurysm, and 8 (6.7%) underwent a heart transplant. Among those with comorbidities, 54 (51.9%) were hypertensive, 22 (21.2%) were diabetic, and 10 (9.6%) had lung diseases. Among those who had complications, 61 (52.6%) had pneumonia, 50 (42.4%) developed renal failure, and 49 (51.0%) had a moderate form of the transient disturbance of gas exchange. Noninvasive ventilation was performed in 53 (44.5%) patients. The death rate was 40.3%, and mortality was higher in the group that did not receive noninvasive ventilation before reintubation (53.5%). Within the reintubated patients who died, the SOFA and APACHE II values were 7.9 ± 3.0 and 16.9 ± 4.5, respectively. Most of the reintubated patients (47.5%) belonged to the high-risk group, EuroSCORE (> 6 points).

**Conclusion:**

The reintubation rate was high, and it was related to worse SOFA, APACHE II and EuroSCORE scores. Mortality was higher in the group that did not receive noninvasive ventilation before reintubation.

## Abstract (pt)

**Objetivo:**

Analisar pacientes em pós-operatório de cirurgia cardíaca que necessitaram de reintubação endotraqueal, e identificar os fatores associados com óbito e seu relacionamento com escores de severidade.

**Métodos:**

Análise retrospectiva de informações referentes a 1.640 pacientes em pós-operatório de cirurgia cardíaca no período entre 2007 e 2015.

**Resultados:**

A taxa de reintubação foi de 7,26%. Dentre os pacientes reintubados, 36 (30,3%) foram submetidos à cirurgia de revascularização miocárdica, 27 (22,7%) à substituição valvar, 25 (21,0%) à correção de um aneurisma e oito (6,7%) a um transplante cardíaco. Dentre os pacientes com comorbidades, 54 (51,9%) eram hipertensos, 22 (21,2%) diabéticos e 10 (9,6%) tinham doença pulmonar. Dentre os pacientes que tiveram complicações, 61 (52,6%) tiveram pneumonia, 50 (42,4%) desenvolveram insuficiência renal e 49 (51,0%) tiveram uma forma moderada de distúrbio transitório da troca gasosa. Foi realizada ventilação não invasiva em 53 (44,5%) pacientes. A taxa de óbitos foi de 40,3%, e a mortalidade foi mais elevada no grupo que não recebeu ventilação não invasiva antes da reintubação (53,5%). Dentre os pacientes reintubados que morreram, os valores do SOFA e do APACHE II foram, respectivamente, de 7,9 ± 3,0 e 16,9 ± 4,5. A maior parte dos pacientes reintubados (47,5%) pertencia ao grupo de risco mais elevado (EuroSCORE > 6 pontos).

**Conclusão:**

A taxa de reintubação foi elevada e se relacionou com o SOFA e o APACHE II mais graves. A mortalidade foi mais elevada no grupo que não recebeu ventilação não invasiva antes da reintubação.

## INTRODUCTION

Since the advent of cardiac surgery in the 1950s, the number of cardiac procedures has grown worldwide.^(1)^ Therefore, it is important to know the factors that can cause complications in the postoperative period and the clinical outcomes of patients undergoing such surgery.^(2)^ The reintubation in this group of patients is related to undesirable consequences because it is associated with higher rates of morbidity and mortality, duration of stay in the intensive care unit (ICU) and hospital costs and resources.^(3,4)^

Thus, we aim to analyze the sample of patients after cardiac surgery, which required reintubation during their stay in the adult ICU of the *Hospital de Clínicas* of *Universidade Estadual de Campinas* (UNICAMP). Additionally, we intend to identify factors associated with death and its relationship with the severity scores of Acute Physiology and Chronic Health Evaluation (APACHE II) in the first 24 hours after ICU admission, Sequential Organ Failure Assessment (SOFA) and European System for Cardiac Operative Risk Evaluation (EuroESCORE).

## METHODS

This is a retrospective and descriptive study based on an analysis of information contained in postoperative databases of the ICU of the *Hospital de Clínicas* of the UNICAMP. The postoperative ICU has 17 beds serving the specialties of cardiac surgery, vascular surgery, and neurosurgery. The professional staff was selected based on the Brazil decree 07, which is specific for ICUs since 2010.^(5)^ The staff was composed of medical residency students in intensive care and students of physical therapy performing continuing education in adult ICUs. The multidisciplinary team care was performed within 24 hours. A single nurse performed the data collection on a daily basis during medical visits using medical records and multidisciplinary team record notes. The scores were calculated daily through applications that are integrated within the database program. All the information was extracted from the database for statistical analysis in a confidential manner to maintain the integrity and privacy of the patients. The research was submitted to the Ethics Committee of the institution and approved under the number 48898915.4.00005404 and registered by Brazilian Registry of Clinical Trials (REBEC) number RBR 6j6fcx.

Data from 1,640 patients undergoing surgery or cardiac procedures were analyzed; these patients had been referred to the postoperative ICU of the specific specialty and required the use of invasive mechanical ventilation by endotracheal tube between May 2007 and April 2015. The study excluded patients under 14 years old, those who were extubated in the operating room, and those who had incomplete data in the database.

All subjects underwent the weaning and extubation protocol developed by the multidisciplinary team of the ICU, which is available in the "Clinics Hospital UNICAMP Adult ICU Procedures Manual." The subjects were considered able to perform a spontaneous breathing trial according to the following criteria: resolution or improvement of the causes of respiratory failure, minute volume ≤ 10 - 15L/min, positive end-expiratory pressure (PEEP) ≤ 5 - 8cmH_2_O, fraction of inspired oxygen (FiO_2_) ≤ 0.4 partial pressure of oxygen/fraction of inspired oxygen (PaO_2_/FiO_2_) > 150mmHg, pH > 7.25, intact respiratory mechanics and hemodynamically stable with low doses of vasoactive drugs. The spontaneous breathing trial was performed in the pressure support mode with a value of 7cmH_2_O and PEEP between 5 and 8cmH_2_O, between 30 minutes and 1 hour. Weaning and extubation failure was indicated by returning to mechanical ventilation in less than 48 hours.^(6)^

For data analysis, subjects were divided into two groups: a group of patients who required reintubation one or more times in the ICU and the other group of patients who were not reintubated during the ICU stay. To analyze the data, reintubated patients in the ICU were divided into 2 groups, namely, the group who died, and those who did not die. The variables related to the regression method that were considered death prognostic predictors were the following: use of non-invasive mechanical ventilation, APACHE II calculation in the first 24 hours after ICU admission, SOFA and EuroSCORE, chronic obstructive pulmonary disease (COPD), age and endocarditis.

We determined the number of individuals who required endotracheal reintubation during the ICU stay and evaluated the demographic characteristics, type of surgery (emergency or elective), indication for surgery (valve replacement, coronary artery bypass, aneurysm repair, heart transplantation), comorbidities (hypertension, diabetes mellitus, arrhythmias, chronic obstructive pulmonary disease), postoperative complications, presence of transient disturbance of gas exchange (TDGE), use of non-invasive ventilation (NIV), mortality and scores APACHE II, SOFA and EuroSCORE of gravity.

The EuroSCORE provides information on risk factors and mortality.^(7)^ The factors related to the patient are age, sex, chronic lung disease, extracardiac arteriopathy, neurological dysfunction, previous cardiac surgery, serum creatinine, active endocarditis, and preoperative critical condition. The factors related to the heart are unstable angina, left ventricular dysfunction, recent myocardial infarction, and pulmonary hypertension. The factors related to the emergency procedure included other surgeries in addition to myocardial revascularization.

### Statistical analyses

A descriptive analysis is presented with tables of frequencies for categorical variables and position and dispersion measures for numeric variables. To compare proportions, we used the chi-square test or Fisher's exact test if necessary. For comparison of numerical measurements between two groups, we used the Mann-Whitney test. To evaluate the factors related to death, we used a logistic regression analysis and univariate and multiple models with Stepwise selection criteria variables. To analyze the relationship between the numerical variables, we Spearman's correlation coefficient. The significance level was set at 5%.

## RESULTS

Of the total 1,640 patients eligible for the study, 119 individuals - 7.3% (95% confidence interval - 95%CI, 6.1 to 8.7) required endotracheal reintubation during their ICU stay. Of this population, 69 (58%) were male, and 50 patients (42%) were female, with a mean age of 59.6 ± 14.4 years. The most common indication for surgery was myocardial revascularization (30.6%), followed by valve replacement (22.7%) and thoracic aortic aneurysm repair (21%). Regarding the severity scores, the average APACHE II and mortality predicted by APACHE II were 15% ± 4.4 and 24.2% ± 11.7 points, respectively. The mean total of SOFA, calculated from the data collected on the first day of ICU admission (SOFA day 1) was 6.9 ± 2.6 points. The average EuroSCORE used for risk stratification in cardiac surgery was 10.4 ± 13.0 points, whichever was the high-risk group (47.5%). The descriptive analysis of the study population is listed in tables 1 and 2.

**Table 1:** Baseline characteristics of patients submitted to cardiac surgery that needed tracheal reintubation (n = 119/1640)

Variables	Total
Sex	
Female (N = 50)	42
Male (N = 69)	58
Age (years)	59.6 ± 14.4
Surgery type	
Elective (N = 86)	72.3
Urgent (N = 33)	27.7
Cause	
Myocardial revascularization (N = 36)	30.3
Valve replacement (N = 27)	22.7
Aneurysm (N = 25)	21.0
Heart transplant (N = 8)	6.7
Myocardial revascularization + valve replacement (N = 12)	10.1
Others* (N = 11)	9.2
Comorbidities	
Arterial hypertension (N = 54)	51.9
Diabetes Mellitus (N = 22)	21.2
Cardiac insufficiency (N = 17)	16.3
Active smoking (N = 20)	19.2
COPD (N = 10)	9.6
Cardiac arrhythmia (N = 10)	9.6

COPD - chronic obstructive pulmonary disease.

* Atrial/ventricular septal defect correction; ventricle aneurysmectomy. Values are expressed as percentage or mean ± standard deviation.

**Table 2:** Clinical outcomes of patients submitted to cardiac surgery that needed tracheal reintubation

Variables	Total
Postoperative complications	
Pneumonia/VAP (N = 61)	52.6
Kidney dysfunction (N = 50)	42.4
AMI (N = 3)	2.5
Endotracheal reintubation in ≤ 48 hours (N = 38)	49.4
Use of NIV in the postoperative period (N = 53)	44.5
Deaths (N = 48)	40.3
APACHE (N = 118)	15.0 ± 4.4
APACHE mortality (N = 118)	24.2 ± 11.7
EuroSCORE (N = 80)	10.4 ± 13.0
SOFA (N = 67)	6.9 ± 2.6
TDGE	
Absent (PaO_2_/FiO_2_ > 300) (N = 11)	11.5
Mild (PaO_2_/FiO_2_: 200 - 300) (N = 28)	29.2
Length of hospital stay (days) N = 119	22.7 ± 19.1

VAP - ventilator-associated pneumonia; AMI - acute myocardial infarction; NIV - noninvasive ventilation; APACHE - Acute Physiology and Chronic Health Evaluation; EuroSCORE - European System for Cardiac Operative Risk Evaluation; SOFA - Sequential Organ Failure Assessment score; TDGE - transient gas exchange disturbance; PaO_2_/FiO_2_ - partial pressure of oxygen/fraction of inspired oxygen ratio. Values are expressed as percentage or mean ± standard deviation.

Of the intubated patients, 48 (40.3%) died. Only Apache, the mortality predicted by APACHE II, the SOFA and the use of NIV were significantly different among the population that eventually died and the people who survived. The other analyzed variables included TDGE, pneumonia, ventilator-associated pneumonia (VAP), arterial hypertension, chronic renal failure, smoking and reintubation in less than 48 hours, and these variables were homogeneous among the populations. The comparative analysis between the patients who died and those who survived is shown in table 3.

**Table 3:** Characteristics of reintubated patients according to survival status

Variables	Death	No death	Total	p value
APACHE	16.9 ± 4.5 (N=47)	14.6 ± 4.2 (N=71)	15.5 ± 4.4 (N=118)	0.01*
APACHE mortality	27.9 ± 12.9 (N=47)	21.7 ± 10.3 (N=71)	24.2 ± 11.7 (N=118)	0.01*
SOFA	7.9 ±3.0 (N=24)	6.3 ± 2.2 (N= 43)	6.9 ± 2.6 (N=67)	0.03*
Use of NIV	31.3 (N=15)	53.5 (N=38)	44.5 (N=53)	0.01†
TDGE				
Absent	11.1 (N=4)	11.7 (N=7)	11.5 (N=11)	0.86‡
Mild	33.3 (N=12)	26.7 (N=16)	29.2 (N=28)	
Moderate	50.0 (N=18)	51.7 (N=31)	51.0 (N=49)	
Severe	5.6 (N=2)	10.0 (N=6)	8.3 (N=8)	
Arterial hypertension	43.9 (N=18)	57.1 (N=36)	51.9 (N=54)	0.19†
Smoking	19.5 (N=8)	19.0 (N=12)	19.2 (N=20)	0.95†
Pneumonia	46.7 (N=21)	56.3 (N=40)	52.6 (N=61)	0.31†
VAP	21.4 (N=9)	26.5 (N=18)	24.5 (N=27)	0.55†
CKF	14.6 (N=6)	7.9 (N=5)	10.6 (N=11)	0.33‡
Reintubation < 48 hours	58.6 (N=17)	43.8 (N=21)	49.4 (N=38)	0.21†
Endocarditis	40.34 (N= 4)	59.66 (N=5)	7.56 (N=9)	0.44

APACHE - Acute Physiology and Chronic Health Evaluation; SOFA - Sequential Organ Failure Assessment score; NIV - noninvasive ventilation; TDGE - transient gas exchange disturbance; VAP - ventilator-associated pneumonia; CKF - chronic kidney failure.

* Mann-Whitney’ test;

† Chi-square test;

‡ Fisher’s exact test. Values are expressed as the mean ± standard deviation or percentage.

Death risk factors were calculated using a univariate logistic regression analysis using the following variables: age, COPD, APACHE, SOFA, NIV and EuroSCORE, endocarditis, VAP and TDGE. These are shown in table 4.

**Table 4:** Univariate logistic regression analysis of risk factors for death

Variables	OR	95%CI	p value
Age	1.00	0.97 - 1.02	0.90
COPD	1.61	0.43 - 5.95	0.47
No use of NIV in the postoperative period	2.53	1.17 - 5.46	0.01
APACHE II mortality	1.04	1.01 - 1.08	0.08
SOFA	1.27	1.03 - 1.57	0.02
EuroSCORE	1.00	0.97 - 1.04	0.65
Endocarditis	1.94	0.49 - 7.66	0.34
VAP	0.67	0.32 - 1.43	0.31
TDGE			
Mild	1.31	0.31 - 5.53	0.71
Moderate	1.01	0.26 - 3.95	0.98
Severe	0.58	0.07 - 4.38	0.6
Acute kidney injury	0.75	0.35 - 1.60	0.46
Time of MV	0.55	0.16 - 1.81	0.32

OR - odds ratio; 95%CI - 95% confidence interval; COPD - chronic obstructive pulmonary disease; NIV - noninvasive ventilation; APACHE - Acute Physiology and Chronic Health Evaluation; SOFA - Sequential Organ Failure Assessment score; EuroSCORE - European System for Cardiac Operative Risk Evaluation; VAP - ventilator-associated pneumonia; TDGE - transient gas exchange disturbance; MV - mechanical ventilation.

The variables that are collectively associated with death are the following: NIV (not using it increases the chance of death 3-fold), and mortality measured by APACHE II (each increased unit in the probability increases the chance of death by 5.7%). These variables are shown in table 5.

**Table 5:** Multivariate analysis of the risk factor for death in the use of noninvasive ventilation and mortality calculated by Acute Physiology and Chronic Health Evaluation

Variables	OR	95%CI	p value
No use of NIV in the postoperative period	3.07	1.10 - 8.54	0.03
APACHE II mortality	1.05	1.01 - 1.1	0.01

OR: odds ratio; 95%CI - 95% confidence interval; NIV - noninvasive ventilation; APACHE - Acute Physiology and Chronic Health Evaluation.

## DISCUSSION

The reintubation rate in this study was 7.26%. It is superior to the rates found in other studies, which range from 3.8% to 6.6%^(8,9)^, and also disagrees with the suggestion that university hospitals tend to have lower rates of reintubation.^(10)^ This result shows that at least in this service, factors other than the presence of trained professionals and protocols for extubation may influence outcomes in this context.

According to Zahoor and Azlina,^(11)^ individuals subjected to valve replacement have a higher risk of needing reintubation compared with patients who underwent coronary artery bypass surgery. An explanation for this finding would be impaired preoperative pulmonary function, which worsens postoperatively, with a fall in complacency. Therefore, it is difficult to accommodate the blood volume of the lungs after correction of the valve defect. In this study, however, the highest reintubation rate was found in patients with coronary artery bypass surgery (30.3%), possibly because this procedure is the most prevalent surgery service.

Smoking and hypertension are risk factors for extubation failure, which consequently leads to prolonged use of invasive mechanical ventilation, need for reintubation and mortality in postoperative cardiac surgery patients.^(10,11)^ In this study, the percentage of smokers and hypertensive subjects showed no significant differences between the death group and the surviving group. Similarly, it was observed that there was no correlation between smoking, chronic obstructive pulmonary disease and reintubation.^(2,12,13)^

Cardiovascular, pulmonary and renal complications are also reported in the literature as factors related to reintubation and greater mechanical ventilation.^(9)^ In the studied population, 2.5% had acute myocardial infarction and more than half (52.6%) of patients had nosocomial pneumonia or VAP. The latter figure is consistent with other authors, who observed a higher incidence of pneumonia in reintubated patients and longer duration of mechanical ventilation. Thus, preventive measures and treatments must be developed and improved to shorten the duration of mechanical ventilation and increase the chances of survival.^(14,15)^

Regarding renal complications, 42.4% developed some degree of renal dysfunction, defined here as serum creatinine > 1.2mg/dL (value from which the SOFA considers renal failure), regardless of whether renal replacement therapy was needed during hospitalization. In the study by Piotto et al., despite the use of serum creatinine value, renal failure was the strongest predictor of the need for prolonged mechanical ventilation.^(16)^ Exarchopoulos et al. noted that SOFA score is one of the most significant predictors of mortality and in-hospital postoperative comorbidities in cardiac surgery patients.^(17)^

Prolonged mechanical ventilation is defined in the literature as the use of invasive mechanical ventilation for more than 48 hours^(16)^ and is closely related to endotracheal reintubation because the factors or complications that led the patient to need new ventilatory support are rarely resolved in less than two days. Thus, the length of stay in the ICU can exceed 2 to 3 weeks,^(18)^ mortality increases by 40% and the effective cost of patients on mechanical ventilation for more than 4 days rises up to 18 times, leading to substantial economic impact.^(19)^ In this work, we have chosen to only study the population submitted to reintubation and therefore subject to all the complications that can prolong the ICU stay; the mean hospital stay was 22.7 ± 19.1 days. Cohen et al. defined reintubation as the increase in postoperative complications and extension in the intensive care unit and hospitalization.^(20)^

Practically all patients who are in the postoperative period of cardiac surgery have some degree of pulmonary dysfunction.^(21,22)^ Factors related to the surgical procedure are involved in the pathogenesis of this problem: the pain caused by drains and the surgical incision creates a restrictive phenomenon that compromises respiratory dynamics, hindering gas exchange and favoring the appearance of atelectasis. The need for blood product transfusions and extracorporeal circulation also affect lung function by deflagrating important inflammatory response.^(23)^

Regarding the transient disturbance of gas exchange, most reintubated patients presented mild (29.2%) and moderate (51.0%) forms. The pathophysiology behind this disorder is summarized in the presence of pro-inflammatory cytokines released by activated leukocytes after getting in contact with non-endothelial surfaces of extracorporeal circulation. Under the influence of these interleukins, a release of oxygen-derived free radicals and proteases that damage the pulmonary microvasculature modify the gas exchange.^(24)^ Rodrigues et al. found similar values with respect to mild (27.7%) and moderate (56.1%) forms of TDGE in patients undergoing cardiac surgery.^(22)^ In the same study, the researchers correlated the APACHE II (12.8 ± 4.2 points) and mortality predicted by APACHE II (17.9 ± 9.5 points) with the presence of severe TDGE (p = 0.0001), suggesting that the APACHE II can also be a predictor of severe TDGE.

The APACHE II is an index used to classify critical patients according to the severity of their condition.^(23)^ The mean APACHE II in this study was 15 ± 4.4 points, and the predicted mortality was 24.2 ± 11.7 points. Similar values were seen in the work by Feijó et al., in which the average APACHE II was 16.5 ± 7.7 points.^(24)^ However, the overestimation of APACHE II may be responsible for the increase in deaths.^(25)^

The overall analysis of the gravity of this study scores (APACHE II, SOFA and EuroSCORE) shows that the studied population is composed predominantly of critically ill patients, which may explain the high rate of reintubation and mortality. Of the patients who were reintubated, 48 (40.3%) died.

In the comparative analysis between the death group and no deaths, the APACHE II variables, mortality predicted by APACHE II and SOFA scores were higher in the group who died. There was a significant relationship between mortality predicted by Apache and risk of death (p = 0.0105), showing that this score can also be used to predict mortality in reintubated patients. Tsaousi et al. found that EuroSCORE appears to confer remarkable prognostic value, almost equivalent to the SOFA score and higher than APACHE II, which are the main risk stratification scores for morbidity and mortality prediction in the cardiac surgery population.^(26)^

The use of noninvasive ventilation (NIV) preceding reintubation was associated with a lower mortality according to our multivariate model adjusted for baseline APACHE II. However, although these results at first suggest a beneficial effect of NIV, alternative explanations are very reasonable, and a causal relationship cannot be established. Unadjusted confounders may be involved. For example, NIV is contraindicated for very sick patients in a coma or with severe shock.

If NIV is indeed beneficial in post-extubation respiratory failure, possible mechanisms might be the fact that the NIV prophylactic in patients undergoing cardiac surgery improves oxygenation rates and reduces the risk of pulmonary complications in the postoperative period.^(27,28)^ In a prospective study of patients after cardiac surgery, Zhu et al. concluded that the use of NIV is associated with a lower reintubation rate (18.8% *versus* 80.9%), lower incidence of VAP (0 *versus* 17%) and the lowest mortality rate (18.8% *versus* 38.3%) compared to conventional treatment.^(29)^ In a meta-analysis study, Olper et al. demonstrated effectiveness of the use of NIV in reducing reintubation in fourteen clinical trials that evaluated the benefits after cardiac surgery.^(30)^ Other studies of respiratory dysfunction in the postoperative period of major surgery, such as solid organ transplantation and lung resections, also suggest that NIV reduces the need for reintubation and mortality.^(31,32)^ According to Jaber et al., NIV prevented reintubation in 67% of patients undergoing abdominal surgery.^(33)^

This is a cohort type study composed of a population that experiences an event that occurred at the same time. The analysis of this study was performed with postoperative cardiac surgery patients who underwent various procedures; however, all had similar APACHE II scores. Additionally, the available data storage system is obsolete for the data analysis, which is a common situation in Brazilian public hospitals.

The study's strengths include the large sample size and correlated variables. If the results were analyzed by a clinical trial, it would have required more time and higher costs.

Due to the large sample in this study, some data could not be captured by the database to record the variables of ventricular function, pulmonary hypertension and extracardiac arteriopathy isolated from EuroSCORE. Therefore, further studies should examine these variables independently to evaluate reintubation and mortality of postoperative cardiac surgery patients.

## CONCLUSION

The reintubation rate in patients undergoing cardiac surgery in our hospital was high. Of the reintubated patients, many died, and this outcome is associated with high APACHE II and SOFA scores. The use of non-invasive ventilation was associated with reduced mortality in reintubated patients. Its use for prophylaxis in patients after cardiac surgery should be considered and enhanced to avoid reintubation and increase the chances of survival.
